# Risk for subsequent infection and mortality after hospitalization among patients with multidrug-resistant gram-negative bacteria colonization or infection

**DOI:** 10.1186/s13756-018-0388-z

**Published:** 2018-07-31

**Authors:** Wen-Pin Tseng, Yee-Chun Chen, Shang-Yu Chen, Shey-Ying Chen, Shan-Chwen Chang

**Affiliations:** 1 Department of Emergency Medicine, National Taiwan University Hospital, College of Medicine, National Taiwan University, No. 7, Zhongshan S. Rd., Zhongzheng Dist., Taipei, 100 Taiwan; 2 Department of Internal Medicine, National Taiwan University Hospital, College of Medicine, National Taiwan University, No. 7, Zhongshan S. Rd., Zhongzheng Dist., Taipei, 100 Taiwan; 3 Center for Infection Control, National Taiwan University Hospital, College of Medicine, National Taiwan University, No. 7, Zhongshan S. Rd., Zhongzheng Dist., Taipei, 100 Taiwan

**Keywords:** Multidrug resistance, Gram-negative bacteria, Colonization, Subsequent infection, Mortality

## Abstract

**Background:**

Risks for subsequent multidrug-resistant gram-negative bacteria (MDRGNB) infection and long-term outcome after hospitalization among patients with MDRGNB colonization remain unknown.

**Methods:**

This observational study enrolled 817 patients who were hospitalized in the study hospital in 2009. We defined MDRGNB as a GNB resistant to at least three different antimicrobial classes. Patients were classified into MDRGNB culture-positive (MDRGNB-CP; 125 patients) and culture-negative (MDRGNB-CN; 692 patients) groups based on the presence or absence of any MDRGNB identified from either active surveillance or clinical cultures during index hospitalization. Subsequent MDRGNB infection and mortality within 12 months after index hospitalization were recorded. We determined the frequency and risk factors for subsequent MDRGNB infection and mortality associated with previous MDRGNB culture status.

**Results:**

In total, 129 patients had at least one subsequent MDRGNB infection (MDRGNB-CP, 48.0%; MDRGNB-CN, 10.0%), and 148 patients died (MDRGNB-CP, 31.2%; MDRGNB-CN, 15.9%) during the follow-up period. MDR *Escherichia coli* and *Acinetobacter baumannii* were the predominant colonization microorganisms; patients with *Proteus mirabilis* and *Pseudomonas aeruginosa* had the highest hazard risk for developing subsequent infection. After controlling for other confounders, MDRGNB-CP during hospitalization independently predicted subsequent MDRGNB infection (hazard ratio [HR], 5.35; 95% confidence interval [CI], 3.72–7.71), all-cause mortality (HR, 2.42; 95% CI, 1.67–3.50), and subsequent MDRGNB infection-associated mortality (HR, 4.88; 95% CI, 2.79–8.52) after hospitalization.

**Conclusions:**

Harboring MDRGNB significantly increases patients’ risk for subsequent MDRGNB infection and mortality after hospitalization, justifying the urgent need for developing effective strategies to prevent and eradicate MDRGNB colonization.

## Key points

Harboring multidrug-resistant gram-negative bacteria (MDRGNB) significantly increases patients’ risk for subsequent infection after hospital discharge. The association between MDRGNB colonization and increased long-term mortality further justifies the need for effective, collaborative strategies to prevent and eradicate MDRGNB colonization.

## Background

The emergence and spread of multidrug-resistant gram-negative bacteria (MDRGNB) have become a major public health threat globally [1, 2]. Infections with MDRGNB are associated with higher hospital cost, prolonged hospitalization, and mortality [3–7]. Acquisition and infection of MDRGNB are common among hospitalized patients, especially for critically ill patients who were vulnerable to high MDRGNB selection pressure following extensive antimicrobial therapy [8–12]. The risk for subsequent infection was significantly higher for hospitalized patients with initial antimicrobial-resistant GNB colonization than patients without colonization [8, 13–15]. Approximately 9.1–39% of inpatients who were initially colonized with various antimicrobial-resistant GNB developed subsequent infection during the same hospital stay [8, 10, 12–14]. Therefore, the effect of initial MDRGNB colonization on the risk for subsequent infection and clinical outcomes among hospitalized patients is well documented, and it significantly influences the recommendations for controlling and treating MDRGNB infections in hospital settings.

In contrast to in-hospital settings, decreased risk for MDRGNB colonization and infection over time following hospital discharge was usually deemed as a matter of course. Although the clearance of MDRGNB colonization of a patient after being away from nosocomial, high-antibiotic pressure environment has been demonstrated [16], prolonged effect from persistent MDRGNB colonization remains a potential contributing factor for subsequent MDRGNB infections and threats on the treatment outcomes of community patients with recent hospitalization history. However, the effects of MDRGNB colonization on subsequent infection and long-term outcome of patients after hospitalization have not been comprehensively explored.

In this study, we used any positive culture for MDRGNB, either from active surveillance cultures on index hospitalization admission or decision-driven clinical cultures during index hospitalization, to identify patients with potential MDRGNB colonization, irrespective of the antibiotic treatment history. Then, we hypothesized that MDRGNB colonization along with certain patient characteristics synergistically affects the risk for subsequent MDRGNB associated infection and long-term mortality after hospital discharge. We also hypothesized that there exists different species-specific and isolation site-specific colonization effects that contribute to the different risk for subsequent MDRGNB infection. Therefore, this study aimed to provide data on the characteristics of subsequent infection pattern and outcome effects associated with prior MDRGNB colonization or infection to help first-line physicians in making treatment decisions for patients with community-onset infection.

## Methods

### Study design, setting, and patients

The National Taiwan University Hospital is a 2200-bed teaching hospital that provides both primary and tertiary care in northern Taiwan. This retrospective study used an adult patient cohort of one prospective study that recruited 995 patients in the emergency department who received active microbiological surveillance cultures for the development a MDRGNB prediction model on patients’ hospital admission (index hospitalization) [17]. The results of active surveillance cultures, which included anterior nares swab, posterior pharyngeal wall (throat) swab, urine, and areas of skin breakdown if presented, and all clinical cultures during index hospitalization of the 995 patients were recorded to determine the MDRGNB colonization status on admission and before hospital discharge. Among the 995 patients, 118 (11.6%) died during hospitalization and 60 (6.0%) did not have any outpatient department (OPD) follow-up record after hospital discharge; hence, they were excluded from this study. Therefore, only 817 patients who survived the index hospitalization discharge and had at least one OPD follow-up record were finally enrolled. We then conducted this observational study to investigate the risk for subsequent MDRGNB infections and mortality in these patients during 12 months after discharge from the index hospitalization (Appendix).

### Definition of MDRGNB and determination of colonization status

We defined MDRGNB as the presence of *Enterobacteriaceae* or glucose non-fermentative gram-negative bacilli (NFGNB) that are resistant to at least three different antimicrobial classes. For *Enterobacteriaceae*, MDR was defined as resistance to at least three classes of the following agents: third- or fourth-generation cephalosporins, aminoglycosides, fluoroquinolones, and ampicillin/sulbactam. For NFGNB, MDR was defined as resistance to at least three classes of the following agents: antipseudomonal cephalosporins (ceftazidime or cefepime), aminoglycosides, fluoroquinolones (levofloxacin or ciprofloxacin), antipseudomonal penicillins (ticarcillin-clavulanic acid or piperacillin-tazobactam), and carbapenem (imipenem or meropenem) [1, 18]. Patients with and without any positive culture for MDRGNB from either active surveillance cultures on admission or decision-driven clinical cultures during index hospitalization were classified as MDRGNB culture-positive (MDRGNB-CP) and MDRGNB culture-negative (MDRGNB-CN) groups, respectively.

### Data collection and information on variables

For all study patients, clinical data, including age, sex, preexisting comorbidities, antibiotic exposure, intensive care unit (ICU) admission, receiving of tracheal intubation, length of hospital stay (LOS), clinical cultures within 1 year prior to index hospitalization, active surveillance cultures on admission, and decision-driven clinical cultures obtained during index hospitalization were prospectively obtained from medical records [17]. Follow-up data 12 months after index hospitalization were retrospectively collected from the hospital and OPD electronic medical records, including repeated hospitalization, subsequent clinical culture results, occurrence of culture-confirmed MDRGNB infection, and mortality.

Several comorbid medical conditions were investigated. Malignancy included either an active malignant solid tumor or hematological disease. Severity of preexisting comorbidities was assessed using a modified Charlson comorbidity score [19, 20]. The diagnosis of MDRGNB infection of a study patient was independently evaluated by two investigators based on clinical, radiographic, and microbiological findings and National Nosocomial Infections Surveillance criteria [21, 22]. A third investigator confirmed and finalized the decision if the two investigators did not agree on the diagnosis of MDRGNB infection. All subsequent MDRGNB infections were described according to the number of days between the onset of infection and the index hospitalization discharge date, source of infection, and causative microorganism. For patients with multiple episodes of subsequent MDRGNB infection during the 12 months follow-up period, only the first episode was analyzed, because the causative microorganism of later episodes of MDRGNB infection could be a new colonization acquired during subsequent hospitalization for the first episode of MDRGNB infection treatment. MDRGNB-associated mortality was defined as MDRGNB bacteremia occurring within 7 days of death or active MDRGNB infection at the time of death [23].

### Statistical analyses

Means (±SD) were calculated for continuous variables, and percentages were used for categorical variables. Independent Student’s *t*-test or Mann–Whitney U test was used to compare continuous variables, and Chi-square or Fisher’s exact test was used to analyze categorical variables. Kaplan–Meier method and log-rank tests were used to compare the cumulative probability of the first episode of subsequent MDRGNB infection and survival after index hospitalization of both the study groups. We screened for variables with *P* values of ≤0.2 using univariate analysis and included these as candidate variables in the multivariate Cox regression model. We then used stepwise selection of these variables to investigate independent risk factors associated with subsequent MDRGNB infection and 1-year survival. The risks for subsequent infection due to the same MDRGNB species or isolation site were presented as hazard risk between patients with positive culture for the indicated MDRGNB species or isolation site and those without any MDRGNB culture during index hospitalization. Data were analyzed using SAS 9.4 (SAS Institute, Cary NC). All *P* values are two sided, and findings with *P* values of < 0.05 were considered statistically significant.

## Results

In total, 817 patients were enrolled in this study, including 125 patients with at least one positive MDRGNB from surveillance (71 patients), clinical (93 patients), or both (39 patients) cultures. The microbiological distribution of MDRGNB species is detailed in Table 1. *Escherichia coli* was the most predominant MDRGNB isolate (37.6% [47/125]), followed by *Acinetobacter baumannii* (25.6% [32/125]), *Pseudomonas aeruginosa* (17.6% [22/125]), and *Klebsiella pneumoniae* (16.8% [21/125]) during the index hospitalization. Demographic and clinical characteristics of the 817 study patients with and without positive MDRGNB culture during index hospitalization are summarized in Table 2. MDRGNB-CP patients were older, had longer index hospitalization LOS, and had higher percentage of prior healthcare-associated exposure or MDRGNB culture before index hospitalization. MDRGNB-CP patients also had a higher percentage of having cardiovascular diseases and a bed-ridden status, presence of a pressure sore or indwelling urinary catheter, receiving ICU care and tracheal intubation, and antibiotic exposure during index hospitalization.

**Table 1:** Bacteriology and Culture Site of Multidrug-resistant Gram-negative Bacteria (MDRGNB) Isolates Identified during Index Hospitalization and Within 1 year after Index Hospitalization Discharge

Bacterial species	MDRGNB culture positive group^a^ (*n* = 125)										MDRGNB culture negative group^b^ (*n* = 692)									
	Overall		Isolation site								Overall		Isolation site							
			Respiratory^c^		Urine		Blood		Others^d^				Respiratory^c^		Urine		Blood		Others^d^	
Index hospitalization^e,f^																				
*Escherichia coli*	47	(37.6)	17	(13.6)	26	(20.8)	4	(3.2)	7	(5.6)	–	–	–	–	–	–	–	–	–	–
*Acinetobacter* species	32	(25.6)	25	(20.0)	7	(5.6)	2	(1.6)	4	(3.2)	–	–	–	–	–	–	–	–	–	–
*Pseudomonas aeruginosa*	22	(17.6)	17	(13.6)	5	(4.0)	0	(0.0)	0	(0.0)	–	–	–	–	–	–	–	–	–	–
*Klebsiella pneumoniae*	21	(16.8)	15	(12.0)	6	(4.8)	1	(0.8)	1	(0.8)	–	–	–	–	–	–	–	–	–	–
*Enterobacter* species	17	(13.6)	11	(8.8)	5	(4.0)	1	(0.8)	3	(2.4)	–	–	–	–	–	–	–	–	–	–
*Proteus mirabilis*	4	(3.2)	3	(2.4)	1	(0.8)	0	(0.0)	0	(0.0)	–	–	–	–	–	–	–	–	–	–
Other bacteria^g^	20	(16.0)	13	(10.4)	2	(1.6)	3	(2.4)	2	(1.6)	–	–	–	–	–	–	–	–	–	–
Subsequent infection^f,h,i^																				
*Escherichia coli*	24	(40.0)	9	(15.0)	13	(21.7)	3	(5.0)	1	(1.7)	13	(18.8)	2	(2.9)	8	(11.6)	3	(4.3)	2	(2.9)
*Acinetobacter* species	15	(25.0)	12	(20.0)	2	(3.3)	1	(1.7)	2	(3.3)	23	(33.3)	22	(31.9)	1	(1.4)	3	(4.3)	0	(0.0)
*Pseudomonas aeruginosa*	12	(20.0)	10	(16.7)	2	(3.3)	0	(0.0)	0	(0.0)	8	(11.6)	6	(8.7)	1	(1.4)	1	(1.4)	0	(0.0)
*Klebsiella pneumoniae*	10	(16.7)	6	(10.0)	4	(6.7)	0	(0.0)	0	(0.0)	14	(20.3)	9	(13.0)	2	(2.9)	3	(4.3)	1	(1.4)
*Enterobacter* species	1	(1.7)	0	(0.0)	0	(0.0)	1	(1.7)	0	(0.0)	7	(11.7)	7	(10.1)	0	(0.0)	0	(0.0)	0	(0.0)
*Proteus mirabilis*	5	(8.3)	3	(5.0)	0	(0.0)	0	(0.0)	2	(3.3)	3	(4.3)	1	(1.4)	0	(0.0)	0	(0.0)	1	(1.4)
Other bacteria	2	(3.3)^j^	0	(0.0)	2	(3.3)	0	(0.0)	0	(0.0)	5	(7.2)^k^	1	(1.4)	1	(1.4)	1	(1.4)	0	(0.0)

^a^Indicates patients with at least 1 positive MDRGNB culture during index hospitalization

^b^Indicates patients without any positive MDRGNB culture during index hospitalization

^c^Including nasal swab, throat swab, and sputum culture

^d^Including axillary or inguinal skin, wound, soft tissue pus, drainage, bile, pleural fluid, ascites, and catheter tip culture

^e^Including surveillance and clinical culture during index hospitalization

^f^One MDR-GNB species could be isolated from different anatomical sites

^g^Including *Serratia marcescens* (4), *Morganella morganii* (2), *Achromobacter xylosoxidans* (2), *Burkholderia cepacia* complex (1), *Citrobacter freundii* (3), *Aeromonas hydrophila* (1), *Aeromonas sobria* (1), *Providencia stuartii* (1), *Sphingomonas paucimobili*s (1), *Klebsiella oxytoca* (1), and unidentified nonfermentative gram-negative bacilli (3)

^h^Within 1 year after discharge from index hospitalization

^i^Diagnosed with positive clinical culture and compatible clinical presentation

^j^Including *Serratia marcescens* (1) and unidentified nonfermentative gram-negative bacilli (1)

^k^Including *Klebsiella oxytoca* (2), *Serratia marcescens* (1), and unidentified non-fermentative gram-negative bacilli (2)

**Table 2:** Clinical Characteristics of Study Patients by the Detection of Multidrug-resistant Gram-negative Bacteria (MDRGNB) in Either Surveillance of Clinical Culture during Index Hospitalization

Characteristics	MDRGNB culture positive group (*n* = 125)		MDRGNB culture negative group (*n* = 692)		*p*-value
Age, mean ± SD (year)	71.5 ± 14.8		64.1 ± 17.7		< 0.001
Male sex	76	(60.8)	399	(57.7)	0.51
LTCF or nursing-home residence	20	(16.0)	18	(2.6)	< 0.001
Long-term hemodialysis	7	(5.6)	17	(2.5)	0.06
Previous MDRGNB isolation^a^	44	(35.2)	27	(3.9)	< 0.001
Co-morbid medical conditions					
Diabetes mellitus	31	(24.8)	188	(27.2)	0.58
Malignancy	28	(22.4)	173	(25.0)	0.53
End-stage renal disease	7	(5.6)	24	(3.5)	0.25
Liver cirrhosis	7	(5.6)	72	(10.4)	0.09
Congestive heart failure	19	(15.2)	59	(8.5)	0.019
COPD	22	(17.6)	60	(8.7)	0.002
Cerebrovascular accident	37	(29.6)	88	(12.7)	< 0.001
Bed-ridden status	61	(48.8)	108	(15.6)	< 0.001
Presence of pressure sore	17	(13.6)	28	(4.1)	< 0.001
Central vascular catheter^b^	13	(10.4)	44	(6.4)	0.10
Long-term urinary catheter	18	(14.4)	37	(5.4)	< 0.001
Charlson comorbidity index, mean ± SD	3.8 ± 2.6		2.8 ± 2.7		< 0.001
High CCI (≥5)	47	(37.6)	174	(25.1)	0.004.
Index hospitalization treatment					
Antibiotics exposure^c^	115	(92.0)	471	(68.1)	< 0.001
3rd or 4th generation cephalosporin	52	(41.6)	156	(22.5)	< 0.001
Ampicillin-sulbactam	42	(33.6)	180	(26.0)	0.08
Piperacillin-tazobactam	41	(32.8)	77	(11.3)	< 0.001
Carbapenem	21	(16.8)	18	(2.6)	< 0.001
Fluoroquinolone	22	(17.6)	51	(7.4)	< 0.001
Aminoglycoside	4	(3.2)	6	(0.9)	0.05
ICU admission	14	(11.2)	21	(3.0)	< 0.001
Receiving of tracheal intubation	14	(11.2)	15	(2.2)	< 0.001
Length of hospital stay, median ± IRQ	19.0 ± 22.0		9.0 ± 12.0		< 0.001

*Abbreviations*: *SD* standard deviation, *MDRGNB* multidrug-resistant gram-negative bacteria, *COPD* chronic obstructive pulmonary disease, *CCI* Charlson comorbidity index, *ICU* intensive care unit, *IRQ* interquartile range

^a^Within 1 year prior to the index hospitalization

^b^Including Port-A catheter, Hickman catheter, permcath catheter, double-lumen catheter, and peripherally inserted central catheter

^c^Including oral and intravenous antibiotic exposure for > 48 h during index hospitalization

**Fig. 1:**
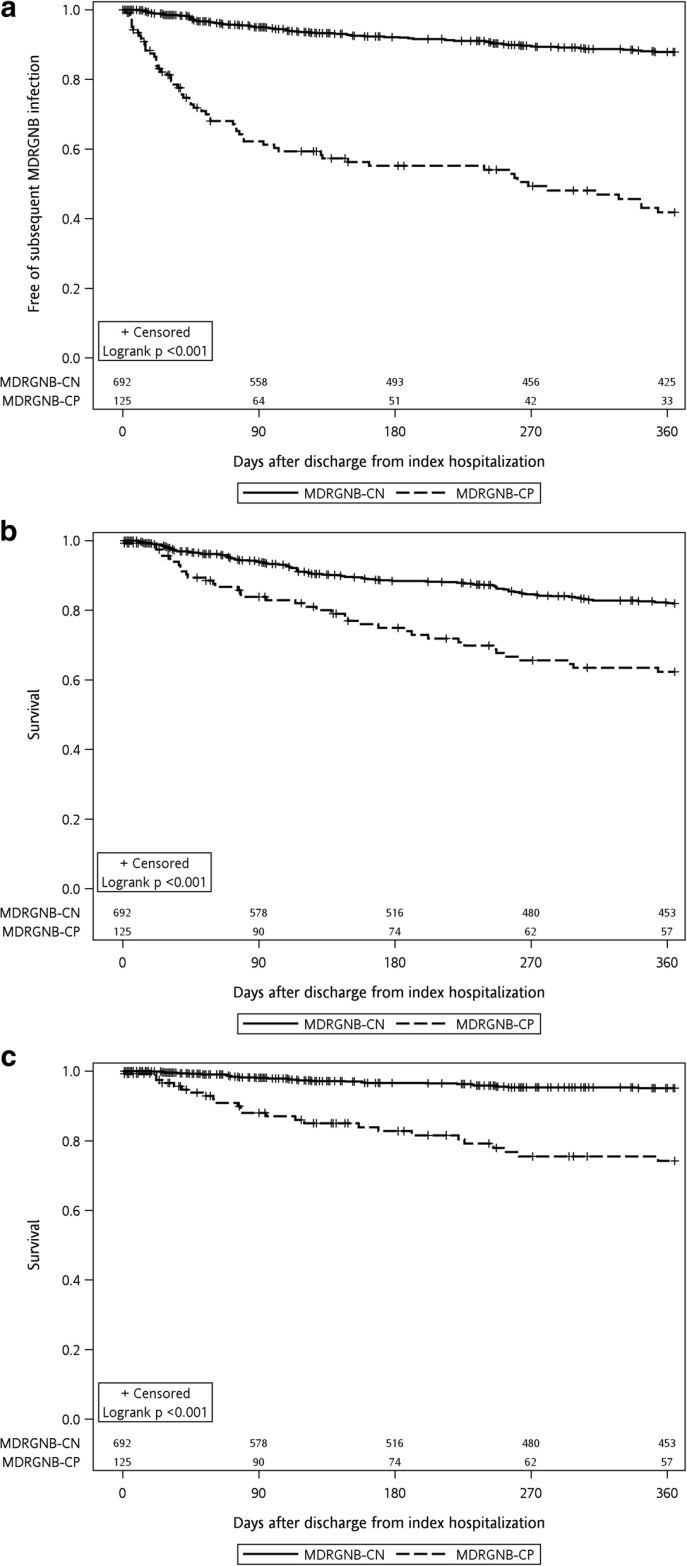
Kaplan–Meier curves at 1 year for **a** subsequent MDRGNB infection, **b** all-cause mortality, and **c** MDRGNB infection-associated mortality after discharge, stratified by index hospitalization MDRGNB culture result. Abbreviations: MDRGNB, multidrug-resistant gram-negative bacteria; MDRGNB-CN, multidrug-resistant gram-negative bacteria culture negative; MDRGNB-CP, multidrug-resistant gram-negative bacteria culture positive

After discharge from index hospitalization, 129 patients (60 MDRGNB-CP and 69 MDRGNB-CN) had at least one episode of subsequent MDRGNB infection during the 12-month follow-up period. The median duration from index hospitalization discharge to the first subsequent MDRGNB infection episode was 74 (range, 2–354) days for all the 129 patients with subsequent infection episodes, 44.5 (range, 2–354) days for the 60 MDRGNB-CP patients, and 108 (range, 10–352) days for the 69 MDRGNB-CN patients. *E. coli* remained the most predominant causative microorganism of subsequent MDR-GNB infections in both MDRGNB-CP and MDRGNB-CN groups (Table 1). Among the 129 patients with subsequent MDRGNB infections, 5 had two concomitant MDRGNB infection foci (respiratory and urinary tracts [4], respiratory tract and intra-abdominal [1]). The lower respiratory tract was the most prevalent primary focus of subsequent MDRGNB infection (66.7% [86/129]), followed by urinary tract (24.8% [32/129]), intra-abdominal area (7.8% [10/129]), other infection foci (2.3% [3/129]), and primary bacteremia (2.3% [3/129]). Among the 60 subsequent MDRGNB infection episodes from the 125 MDRGNB-CP patients, 41 (68.3%) were caused by the same MDR bacterial species, 43 (71.7%) had the infection site concordant to prior MDRGNB culture site, and 33 (55.0%) had the causative MDR bacterial species and culture site concordant to prior MDRGNB culture during index hospitalization. MDRGNB-CP patients had significantly higher cumulative probability of developing subsequent MDRGNB infection than MDRGNB-CN patients (log-rank test, *P* < 0.001) (Fig. 1a). Multivariate Cox regression analysis showed the positive MDRGNB culture during index hospitalization was an independent predictor for subsequent MDRGNB infection (hazard ratio [HR], 5.35; 95% confidence interval [CI], 3.72–7.71), after controlling other potential confounders, such as age (HR, 1.02; 95% CI, 1.01–1.03), malignancy (HR, 1.66; 95% CI, 1.12–2.47), congestive heart failure (HR, 1.70; 95% CI, 1.08–2.68), chronic obstructive pulmonary disease (HR, 1.60; 95% CI, 1.03–2.48), and antibiotics exposure during index hospitalization (HR, 2.20; 95% CI, 1.25–3.90) (Table 3).

**Table 3:** Univariate and Multivariate Cox Regression Analysis for the Risk for Subsequent Multidrug-resistant Gram-negative Bacteria (MDRGNB) Infection after Hospital Discharge

	Univariate analysis			Multivariate analysis		
	HR	(95% CI)	*p*-value	HR	(95% CI)	*p*-value
Age (per year increase)	1.03	(1.02–1.05)	< 0.001	1.02	(1.01–1.03)	0.007
Male sex	0.97	(0.68–1.37)	0.84	–	–	–
LTCF residence	2.59	(1.43–4.70)	0.002	–	–	–
Long-term hemodialysis	1.30	(0.53–3.19)	0.56	–	–	–
Diabetes mellitus	1.13	(0.77–1.64)	0.53	–	–	–
Malignancy	1.41	(0.95–2.09)	0.09	1.66	(1.12–2.47)	0.012
End-stage renal disease	0.96	(0.39–2.34)	0.93	–	–	–
Liver cirrhosis	0.65	(0.32–1.33)	0.24	–	–	–
Congestive heart failure	2.79	(1.83–4.27)	< 0.001	1.70	(1.08–2.68)	0.021
COPD	2.73	(1.80–4.15)	< 0.001	1.60	(1.03–2.48)	0.038
Cerebrovascular accident	1.75	(1.17–2.64)	0.007	–	–	–
Presence of pressure sore	2.32	(1.31–4.12)	0.004	–	–	–
Central vascular catheter^a,b^	1.83	(1.01–3.32)	0.046	–	–	–
Long-term urinary catheter^b^	1.72	(1.00–3.00)	0.05	–	–	–
Antibiotics exposure^c^	3.54	(2.03–6.17)	< 0.001	2.20	(1.25–3.90)	0.007
Intensive care unit admission^c^	1.26	(0.59–2.70)	0.55	–	–	–
Receiving tracheal intubation^c^	2.39	(1.25–4.56)	0.008	–	–	–
MDRGNB culture positive^c,d^	7.19	(5.08–10.19)	< 0.001	5.35	(3.72–7.71)	< 0.001

*Abbreviations*: *LTCF* long-term care facility, *COPD* chronic obstructive pulmonary disease

^a^Includes Port-A catheter, Hickman catheter, permcath catheter, double-lumen catheter, and peripherally inserted central catheter

^b^At the time of index hospitalization discharge

^c^During index hospitalization

^d^Including positive MDR-GNB culture from either surveillance or clinical culture of any anatomical site

Analysis for species-specific risk for subsequent infection after index hospitalization discharge showed that the hazard risk in patients with initial MDR *Proteus mirabilis* culture was higher (HR, 231.29; 95% CI, 32.10–1666.31) than those without any MDRGNB culture, followed by MDR *P. aeruginosa* (HR, 56.99; 95% CI, 21.53–150.88), MDR *E. coli* (HR, 36.83; 95% CI, 17.59–77.14), MDR *K. pneumoniae* (HR, 15.83; 95% CI, 5.17–48.48), and MDR *A. baumannii* (HR, 15.25; 95% CI, 7.58–30.69). With respect to isolation site, previous MDRGNB culture from urinary tract had the highest risk for subsequent MDRGNB urinary tract infection after discharge (HR, 18.45; 95% CI, 8.25–41.27) (Table 4).

**Table 4:** Species-Specific Risk for Subsequent Infection with Causative Multidrug-resistant Gram-negative Bacteria (MDRGNB) or Infection Site Identical to Prior MDRGNB Culture during Index Hospitalization

	Subsequent MDR-GNB infection of concordant species or isolation site		
	Hazard Ratio	(95% C.I.)^a^	*P* value
Initial MDRGNB species			
*Proteus mirabilis*	231.29	(32.10–1666.31)	< 0.001
*Pseudomonas aeruginosa*	56.99	(21.53–150.88)	< 0.001
*Escherichia coli*	36.83	(17.59–77.14)	< 0.001
*Klebsiella pneumoniae*	15.83	(5.17–48.48)	< 0.001
*Acinetobacter* species	15.25	(7.58–30.69)	< 0.001
*Enterobacter* species	5.83	(0.73–46.62)	0.10
Initial MDRGNB isolation site			
Urine	18.45	(8.25–41.27)	< 0.001
Respiratory tract	10.52	(6.81–16.26)	< 0.001
Blood	8.92	(1.14–69.83)	0.037

^a^Calculated as hazard risk between patients with positive culture for the indicated MDRGNB species or isolation site and those without any MDRGNB culture during index hospitalization

A total of 148 in hospital mortality events were observed during the 12-month follow-up period after discharge from index hospitalization; 53 (35.8%) of them were associated with subsequent MDRGNB infections. MDRGNB-CP patients had significantly lower cumulative probability of survival than MDRGNB-CN patients, irrespective of all-cause or subsequent MDRGNB infection-associated mortality (both log-rank tests, *P* < 0.001) (Fig. 1b and c). By Cox regression analysis, MDRGNB-CP during index hospitalization was an independent predictor for all-cause mortality (HR, 2.42; 95% CI, 1.67–3.50) and subsequent MDRGNB infection-associated mortality (HR, 4.88; 95% CI, 2.79–8.52), after controlling other potential confounders, such as age, malignancy, high Charlson comorbidity index, and long-term indwelling urinary catheter (Table 5).

**Table 5:** Univariate and multivariate cox regression analysis for the risk for mortality after discharge from index hospitalization

	All-cause mortality						MDRGNB infection-associated mortality					
	Univariate analysis			Multivariate analysis			Univariate analysis			Multivariate analysis		
	HR	(95% CI)	*p*-value	HR	(95% CI)	*p*-value	HR	(95% CI)	*p*-value	HR	(95% CI)	*p*-value
Age (per year increase)	1.02	(1.01–1.03)	0.001	1.02	(1.01–1.03)	0.006	1.04	(1.02–1.06)	< 0.001	1.03	(1.01–1.05)	0.008
Male sex	1.34	(0.96–1.88)	0.09	–	–	–	1.24	(0.71–2.16)	0.45	–	–	–
LTCF residence	1.33	(0.65–2.71)	0.43	–	–	–	2.37	(0.94–5.95)	0.07	–	–	–
Long-term hemodialysis	1.10	(0.45–2.68)	0.84	–	–	–	1.86	(0.58–5.96)	0.30	–	–	–
Diabetes mellitus	0.89	(0.62–1.29)	0.89	–	–	–	0.93	(0.51–1.72)	0.82	–	–	–
Malignancy	6.28	(4.52–8.73)	< 0.001	5.30	(3.73–7.53)	< 0.001	2.32	(1.32–4.07)	0.004	2.34	(1.28–4.26)	0.006
End-stage renal disease	0.82	(0.34–2.00)	0.82	–	–	–	1.40	(0.44–4.48)	0.57	–	–	–
Liver cirrhosis	1.65	(1.04–2.61)	0.034	–	–	–	0.81	(0.29–2.23)	0.68	–	–	–
Congestive heart failure	1.25	(0.74–2.10)	0.41	–	–	–	2.38	(1.20–4.73)	0.014	–	–	–
COPD	1.40	(0.88–2.25)	0.16	–	–	–	2.62	(1.38–4.99)	0.003	–	–	–
Cerebrovascular accident	0.90	(0.57–1.43)	0.65	–	–	–	1.76	(0.94–3.28)	0.08	–	–	–
High CCI (≥5)	3.33	(2.41–4.60)	< 0.001	1.80	(1.27–2.55)	< 0.001	3.56	(2.08–6.12)	< 0.001	1.96	(1.10–3.49)	0.022
Presence of pressure sore	1.98	(1.13–3.41)	0.016	–	–	–	1.93	(0.77–4.85)	0.16	–	–	–
Central vascular catheter^a,b^	4.07	(2.66–6.22)	< 0.001	–	–	–	2.80	(1.26–6.21)	0.012	–	–	–
Long-term urinary catheter^b^	0.35	(0.13–0.94)	0.037	0.29	(0.11–0.80)	0.017	0.49	(0.12–2.02)	0.33	–	–	–
Antibiotics exposure^c^	1.70	(1.14–2.56)	0.010	–	–	–	3.96	(1.58–9.95)	0.003	–	–	–
MDRGNB culture positive^c,d^	2.40	(1.66–3.46)	< 0.001	2.42	(1.67–3.50)	< 0.001	5.91	(3.44–10.14)	< 0.001	4.88	(2.79–8.52)	< 0.001

*Abbreviations*: *MDRGNB* multidrug-resistant gram-negative bacteria, *LTCF* long-term care facility, *COPD* chronic obstructive pulmonary disease, *CCI* Charlson comorbidity index

^a^Including port-A catheter, Hickman catheter, permcath catheter, double-lumen catheter, and peripherally inserted central catheter

^b^At the time of index hospitalization discharge

^c^During index hospitalization

^d^Including positive MDRGNB culture from either surveillance or clinical culture of any anatomical site

We further divided our MDRGNB-CP patients into 2 subgroups: MDRGNB surveillance culture positive only patients and MDRGNB clinical culture positive patients (all patients with positive MDRGNB clinical culture, including those with both positive MDRGNB clinical and surveillance culture). After controlling for potential confounders, the risk for subsequent MDRGNB infection were both significantly higher in the MDRGNB surveillance culture positive only patients (HR, 4.63; 95% CI, 2.36–9.10) and MDRGNB clinical culture positive patients (HR, 5.56; 95% CI, 3.77–8.20), compared to that of MDRGNB-CN patients. The higher risk for all-cause mortality and subsequent MDRGNB infection-associated mortality after index hospitalization, compared to those of MDRGNB-CN patients, remained significant for the MDRGNB surveillance culture only patients (all-cause mortality, HR, 2.29; 95% CI, 1.19–4.41; subsequent MDRGNB infection-associated mortality, HR, 3.44; 95% CI, 1.20–9.90) and for the MDRGNB clinical culture positive patients (all-cause mortality, HR, 2.46; 95% CI, 1.62–3.74; subsequent MDRGNB infection-associated mortality, 5.33; 95% CI, 2.96–9.60).

## Discussion

This study assessed the effects of prior MDRGNB colonization on subsequent infection risk and long-term outcome after hospital discharge and yielded three major findings. First, even in an environment that is expected to be free of or with low antibiotic selection pressure, the effect of previous MDRGNB colonization on the risk for subsequent MDRGNB infection remains significant and prolonged. Second, species-specific and isolation site-specific risks for the occurrence of subsequent MDRGNB infection were observed. The risk for subsequent infection was especially high for *P. mirabilis* and *P. aeruginosa* or genitourinary tract as colonization microorganisms or anatomical site, respectively. Third, prior MDRGNB colonization history independently predicted long-term survival of patients after hospitalization and directly contributed for more than one-third of late mortality. These findings are novel and important for first-line physicians to prepare for appropriate empirical antibiotics and timely infection control intervention in the treatment of previously hospitalized patients with community-onset infection.

Colonization and infection of antimicrobial-resistant bacteria are common in hospitalized patients and introduce significant risks for short-term morbidity and mortality. The effects of prolonged colonization and subsequent infections after hospital discharge should be comprehensively evaluated; however, only methicillin-resistant *Staphylococcus aureus* (MRSA) and vancomycin-resistant *Enterococcus* were previously reported [23–26]. A retrospective study by Huang et al. found that 17.4% (31/178) of patients with newly identified MRSA colonization or infection developed subsequent MRSA infections that were first manifested after discharge from the index hospitalization during an 18-month follow-up period [24]. In the current study, we demonstrated that 48% (60/125) of MDRGNB colonized or infected patients developed subsequent MDRGNB infections in a 12-month follow-up period, with more than half of these subsequent infections caused by the same MDR bacterial species and culture site. The risk for subsequent MDRGNB infection was especially high within 3 months after hospital discharge. Furthermore, our study found that patients harboring MDRGNB was an independent predictor for subsequent mortality after discharge from index hospitalization. Because previous studies never evaluate post-discharge events for patients with positive MDRGNB culture during hospitalization, the effect of MDRGNB on community disease burden and patient outcome were therefore underestimated. This is the first study that demonstrated the prolonged effect of prior acquisition of antimicrobial-resistant bacteria on subsequent infections and mortality after initial hospitalization, with not only gram-positive bacteria but also GNB. As the prevalence of MDRGNB is continuously increasing in community and hospital environments [1, 2, 27, 28], our study findings highlight the urgent need for effective approaches to prevent the spread of MDRGNB in hospitals and to mitigate the MDRGNB colonization burden after hospitalization.

*E. coli* and *A. baumannii* were the most prevalent MDR species identified from patients during index hospitalization and at subsequent infections in this study. However, species-specific hazard risks for developing subsequent infection were higher for *P. mirabilis* and *P. aeruginosa* than those for *E. coli* and *A. baumannii*. This finding is important and deserves further exploration. A previous study by O’Fallone et al., which serial rectal surveillance cultures were obtained every 3–4 weeks from 33 elderly residents of a long-term care facility, found that multidrug-resistant *P. mirabilis* had significantly lower clearance rate (6.7%) and longer colonization duration than other non-*Proteus* MDR species [16]. However, they did not evaluate the effects from persistent colonization of *P. mirabilis* on the risk for subsequent infection. Our study results, by demonstrating the differential risk for subsequent infection after acquisition of different MDRGNB species, suggest the positive correlation between colonization persistence and risk for subsequent infection after hospital discharge. Further studies that explore the contributing factors for persistent MDRGNB colonization should be conducted to design effective decolonization strategies from infection control and prevention perspectives [29–31].

Though patients identified as harboring MDRGNB had significantly higher risk for developing subsequent MDRGNB infections, more than half of all subsequent MDRGNB infections occurred in patients who were not positive for MDRGNB in either initial surveillance cultures on admission or clinical cultures during hospitalization. These patients might have new MDRGNB colonization but were not detected by decision-driven clinical cultures during index hospitalization. This indicates the limitation of using clinical cultures alone in identifying patients with MDRGNB colonization. Other clinical characteristics, including old age, comorbid illnesses, and prior antibiotics exposure, also help clinicians in early suspicion of patients at risk for subsequent MDRGNB infections [10, 12, 13]. Clinicians should incorporate these data to guide their decision on using appropriate empirical antimicrobial therapy for patients with prior healthcare-associated exposure risk.

This study has several limitations. First, this was a single-center study. Thus, generalization from our findings requires further confirmation. Second, outcome data after index hospitalization were retrospectively collected and are therefore subject to information bias. Third, because not all study patients had completed a 12-month follow-up at our hospital, subsequent MDRGNB infection and mortality event that occurred elsewhere would not have been detected. Fourth, because the gastrointestinal tract is an important anatomical site harboring antimicrobial-resistant *Enterobacteriaceae* and *P. aeruginosa* [16, 32, 33], the lack of perianal swab cultures in active microbiological surveillance may have caused the underestimation of the MDR-GNB colonization rate and may have introduced an information bias and misclassification of study groups. Finally, we did not perform genotypic analysis for the 41 subsequent MDRGNB infection episodes that were caused by the same MDR bacterial species identified during index hospitalization. Therefore, the genotypic concordance between index hospitalization and subsequent infection isolates could not be confirmed.

## Conclusions

The prolonged effects of prior MDRGNB colonization or infection significantly increase the risk for subsequent MDRGNB infection and mortality after hospitalization. *P. mirabilis* and *P. aeruginosa* as causative microorganisms or urinary tract as colonization site is a serious concern. Accurate assessment of the risk for MDRGNB-associated sequelae requires prolonged follow-up after discharge. Furthermore, studies aiming at the prevention of MDRGNB acquisition and eradication of colonization to reduce the prolonged effect of MDRGNB colonization are imperative.

## Figures and Tables

**Fig. 2:**
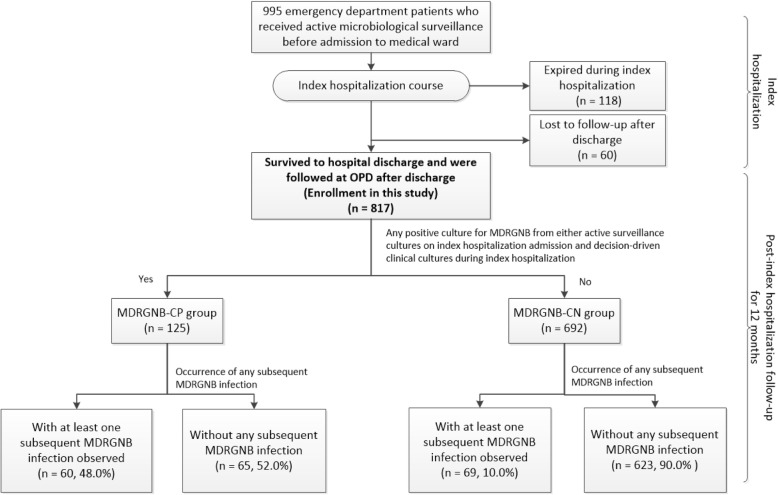
Patient enrollment and classification flowchart. Abbreviations: OPD, outpatient department; MDRGNB, multidrug-resistant gram-negative bacteria; MDRGNB-CP, multidrug-resistant gram-negative bacteria culture positive; MDRGNB-CN, multidrug-resistant gram-negative bacteria culture negative

## Abbreviations

HR: Hazard ratio
ICU: Intensive care unit
LOS: Length of hospital stay
MDRGNB: Multidrug-resistant gram-negative bacteria
MDRGNB-CN: Multidrug-resistant gram-negative bacteria culture negative
MDRGNB-CP: Multidrug-resistant gram-negative bacteria culture positive
NFGNB: Non-fermentative gram-negative bacilli
OPD: Outpatient Department

## References

[1] Pop-Vicas AE, D’Agata EM (2005). The rising influx of multidrug-resistant gram-negative bacilli into a tertiary care hospital. Clin Infect Dis.

[2] Bertrand X, Dowzicky MJ (2012). Antimicrobial susceptibility among gram-negative isolates collected from intensive care units in North America, Europe, the Asia-Pacific rim, Latin America, the Middle East, and Africa between 2004 and 2009 as part of the Tigecycline evaluation and surveillance trial. Clin Ther.

[3] Cosgrove SE (2006). The relationship between antimicrobial resistance and patient outcomes: mortality, length of hospital stay, and health care costs. Clin Infect Dis.

[4] Giske CG, Monnet DL, Cars O, Carmeli Y, ReAct-Action on Antibiotic Resistance (2008). Clinical and economic impact of common multidrug-resistant gram-negative bacilli. Antimicrob Agents Chemother.

[5] Gudiol C, Tubau F, Calatayud L (2011). Bacteraemia due to multidrug-resistant gram-negative bacilli in cancer patients: risk factors, antibiotic therapy and outcomes. J Antimicrob Chemother.

[6] Lye DC, Earnest A, Ling ML (2012). The impact of multidrug resistance in healthcare-associated and nosocomial gram-negative bacteraemia on mortality and length of stay: cohort study. Clin Microbiol Infect.

[7] Vardakas KZ, Rafailidis PI, Konstantelias AA, Falagas ME (2013). Predictors of mortality in patients with infections due to multi-drug resistant gram negative bacteria: the study, the patient, the bug or the drug?. J Inf Secur.

[8] Reddy P, Malczynski M, Obias A (2007). Screening for extended-spectrum beta-lactamase producing Enterobacteriaceae among high-risk patients and rates of subsequent bacteremia. Clin Infect Dis.

[9] Papadomichelakis E, Kontopidou F, Antoniadou A (2008). Screening for resistant gram-negative microorganisms to guide empirical therapy of subsequent infection. Intensive Care Med.

[10] Borer A, Saidel-Odes L, Eskira S (2012). Risk factors for developing clinical infection with carbapenem-resistant *Klebsiella pneumoniae* in hospital patients initially only colonized with carbapenem-resistant *K pneumoniae*. Am J Infect Control.

[11] Hess AS, Kleinberg M, Sorkin JD (2014). Prior colonization is associated increased with increased risk of antibiotic-resistant gram-negative bacteremia in cancer patients. Diagn Microbiol Infect Dis.

[12] Akturk H, Sutcu M, Somer A (2016). Carbapenem-resistant Klebsiella pneumoniae colonization in pediatric and neonatal intensive care units: risk factors for progression to infection. Braz J Infect Dis.

[13] Gómez-Zorrilla S, Camoez M, Tubau F (2015). Prospective observational study of prior rectal colonization status as a predictor for subsequent development of Pseudomonas aeruginosa clinical infections. Antimicrob Agents Chemother.

[14] Harris AD, Jackson SS, Robinson G (2016). Pseudomonas aeruginosa colonization in the intensive care unit: prevalence, risk factors, and clinical outcomes. Infect Control Hosp Epidemiol.

[15] Detsis M, Karanika S, Mylonakis E (2017). ICU acquisition rate, risk factors, and clinical significance of digestive tract colonization with extended-spectrum beta-lactamase-producing Enterobacteriaceae: a systematic review and meta-analysis. Crit Care Med.

[16] O’Fallon E, Gautam S, D’Agata EM (2009). Colonization with multidrug-resistant gram-negative bacteria: prolonged duration and frequent cocolonization. Clin Infect Dis.

[17] Tseng WP, Chen YC, Yang BJ (2017). Predicting multidrug-resistant gram-negative bacterial colonization and associated infection on hospital admission. Infect Control Hosp Epidemiol.

[18] Siegel JD, Rhinehart E, Jackson M, Chiarello L (2007). Management of multidrug-resistant organisms in health care settings, 2006. Am J Infect Control.

[19] Charlson ME, Pompei P, Ales KL, MacKenzie CR (1987). A new method of classifying prognostic comorbidity in longitudinal studies: development and validation. J Chronic Dis.

[20] Schneeweiss S, Wang PS, Avorn J, Glynn RJ (2003). Improved comorbidity adjustment for predicting mortality in Medicare populations. Health Serv Res.

[21] Garner JS, Jarvis WR, Emori TG, Horan TC, Hughes JM (1988). CDC definitions for nosocomial infections, 1988. Am J Infect Control.

[22] Emori TG, Culver DH, Horan TC (1991). National Nosocomial Infections Surveillance System (NNIS): description of surveillance methods. Am J Infect Control.

[23] Datta R, Huang SS (2008). Risk of infection and death due to methicillin-resistant *Staphylococcus aureus* in long-term carriers. Clin Infect Dis.

[24] Huang SS, Platt R (2003). Risk of methicillin-resistant *Staphylococcus aureus* infection after previous infection or colonization. Clin Infect Dis.

[25] Byers KE, Anglim AM, Anneski CJ, Farr BM (2002). Duration of colonization with vancomycin-resistant *Enterococcus*. Infect Control Hosp Epidemiol.

[26] Shenoy ES, Paras ML, Noubary F, Walensky RP, Hooper DC (2014). Natural history of colonization with methicillin-resistant *Staphylococcus aureus* (MRSA) and vancomycin-resistant *Enterococcus* (VRE): a systematic review. BMC Infect Dis.

[27] Ben-Ami R, Rodríguez-Baño J, Arslan H (2009). A multinational survey of risk factors for infection with extended-spectrum beta-lactamase-producing Enterobacteriaceae in nonhospitalized patients. Clin Infect Dis.

[28] Chung DR, Song JH, Kim SH (2011). High prevalence of multidrug-resistant nonfermenters in hospital-acquired pneumonia in Asia. Am J Respir Crit Care Med.

[29] Septimus EJ, Schweizer ML (2016). Decolonization in prevention of health care-associated infections. Clin Microbiol Rev.

[30] Davido B, Batista R, Michelon H (2017). Is faecal microbiota transplantation an option to eradicate highly drug-resistant enteric bacteria carriage?. J Hosp Infect.

[31] Teerawattanapong N, Kengkla K, Dilokthornsakul P, Saokaew S, Apisarnthanarak A, Chaiyakunapruk N (2017). Prevention and control of multidrug-resistant gram-negative bacteria in adult intensive care units: a systematic review and network meta-analysis. Clin Infect Dis.

[32] Villar HE, Baserni MN, Jugo MB (2013). Faecal carriage of ESBL-producing *Enterobacteriaceae* and carbapenem-resistant gram-negative bacilli in community settings. J Infect Dev Ctries.

[33] Wilson AP, Livermore DM, Otter JA (2016). Prevention and control of multi-drug-resistant gram-negative bacteria: recommendations from a joint working party. J Hosp Infect.

